# Characterization of the Key Aroma Compounds of Three Kinds of Chinese Representative Black Tea and Elucidation of the Perceptual Interactions of Methyl Salicylate and Floral Odorants

**DOI:** 10.3390/molecules27051631

**Published:** 2022-03-01

**Authors:** Yunwei Niu, Yiwei Ma, Zuobing Xiao, Jiancai Zhu, Wen Xiong, Feng Chen

**Affiliations:** 1 School of Perfume and Aroma Technology, Shanghai Institute of Technology, Shanghai 201418, China; nyw@sit.edu.cn (Y.N.); 13122028951@163.com (Y.M.); niuge211@sina.com (Z.X.); zjc@sit.edu.cn (J.Z.); 2 School of Agriculture and Biology, Shanghai Jiao Tong University, Shanghai 200240, China; 3 China Tobacco Yunnan Industrial Co., Ltd., Kunming 650231, China; 4 Department of Food, Nutrition and Packaging Sciences, Clemson University, Clemson, SC 29634, USA; fchen@clemson.edu

**Keywords:** black tea, electronic nose, perceptual interaction, headspace, SPME, SAFE

## Abstract

Jinjunmei (JJM), Keemun (KM), and Dianhong (DH) are the representative black teas in China, and they have always been favored by consumers. In this study, we aim to obtain the aroma characteristic information of volatile components in black tea samples through headspace solid-phase microextraction (HS-SPME), solvent-assisted flavor evaporation (SAFE), and gas chromatography-mass spectrometry combined with gas chromatography-olfactometry technology. The results showed that 70 compounds including α-methylbenzyl alcohol (isomer of β-phenylethanol) were identified as odorants. Among them, 39 compounds such as linalool and geraniol showed a high degree of aroma contribution. Furthermore, the Feller’s additive model was used to explore the perceptual interactions among the methyl salicylate and the floral compounds (10 groups): five groups of binary compounds showed masking effect after mixing, one group showed additive effect, and four groups showed synergistic effect. The ratio (R) was compared with the aroma index (n) of Steven’s law, which found a high-fitness exponential relationship. The results of this study help to provide additional and new theoretical guidance for improving the aroma quality of black tea.

## 1. Introduction

Tea (*Camellia sinensis* L.) and tea beverages are becoming more and more popular in the world because of their unique flavor and taste. It has been reported that drinking tea makes people cheerful, and also has many potential health benefits, which keeps tea consumption always at a high level [1,2]. As a fully fermented tea, black tea has a richer flavor than other teas. According to its aroma characteristics, it can be divided into honey-sweet-flavored black tea, fruit-flavored black tea and floral-flavored black tea [3].

Aroma is an important indicator of tea quality. Gas chromatography-mass spectrometry (GC-MS) can achieve the purpose of effectively separating and identifying volatile compounds [4]. There are many ways to extract the aroma of tea, such as headspace solid-phase microextraction (HS-SPME) [5], stir bar sorptive extraction (SBSE) [6], solvent-assisted flavor evaporation (SAFE) [7], and simultaneous distillation extraction (SDE) [8]. These aroma extraction methods have their advantages and disadvantages: HS-SPME can quickly extract volatile compounds, but is not particularly effective for some compounds [9]; SBSE coating has low selectivity, and it is difficult to meet the analysis requirements of complex systems; experimental results may be affected by high temperature during the SDE process [7]; and trace compounds could be extracted more accurately by SAFE [10]. So far, more than 600 volatile compounds have been detected in black tea (including black tea beverages) [11]. Therefore, SAFE combined with SPME was used for the analysis of the aroma compounds of black tea.

There have been many studies on the aroma of black tea, but systematic comparisons of several famous black tea aromas in China are rarely reported. “Qun Fang Zui” Keemun (KM), the “The Treasure of Tea” Jinjunmei (JJM), and the “Flower of Famous Tea” Dianhong (DH) black teas can be regarded as representatives of Chinese black tea. In addition, the floral fragrance in the tea is particularly important. Some scented teas such as jasmine tea have become more and more popular in recent years. This makes it attractive to enhance the floral aroma of black tea. Previous studies have pointed out that the interactions between two aroma compounds include no effect, additive effect, synergistic effect, and masking effect [12]. In recent years, this has gradually attracted more and more attention, and studies have begun to pay attention to the sensory interactions among flavor compounds [13,14]. The research has focused on the identification of aroma compounds, and it is very meaningful to study the interactions among aroma components and their contribution to the aroma of black tea.

In this study, GC-MS combined with gas chromatography-olfactometry (GC-O) were used to identify volatile compounds, and the odor activity value (OAV) was calculated. The OAV is determined by the concentration and odor threshold value, which can show a relative aroma contribution [15]. In addition, an aroma extract dilution analysis (AEDA) was performed to determine flavor dilution (FD) to fully identify key aroma compounds. Furthermore, the perceptual interactions of 10 key floral compounds with the methyl salicylate (MeSA) as the most important ester in black tea were analyzed. Then, the application of Steven’s law in aroma interaction was explored.

As far as we know, there is no research which has systematically compared the aromas of several famous Chinese black teas and deeply explored the perceptual interactions among aroma compounds. In this study, we aim to identify the key aroma compounds of JJM, KM, and DH, three representative Chinese black teas, through the combination of SAFE and HS-SPME, and analyze the similarities and differences of their aroma composition. On this basis, we explore the perceptual interactions among MeSA and floral compounds. The results of this study can improve the flavor of black tea beverages and provide more theoretical support for future black tea flavor innovation.

## 2. Results and Discussion

### 2.1. Key Aroma Compounds Identified of Three Black Teas

In order to completely identify the key aroma compounds in black tea, HS-SPME and SAFE are used in combination with GC-O. A total of 70 volatile aroma compounds in three kinds of black tea were identified, which are listed in Table 1. Among them, JJM (58), DH (49), and KM (62) are expressed by the aroma quality and aroma intensity (AI), or flavor dilution factor (FD) that can be smelled respectively, to clearly show its contribution to the overall aroma of black tea.

#### 2.1.1. Aroma Compounds Identified by SAFE/HS-SPME

For the three kinds of black tea, SAFE and HS-SPME were used as two methods with different extraction principles to recover volatile compounds and obtain relatively complete results [16]. In the experimental results, 65 aromatic compounds were identified by HS-SPME, and 59 aromatic compounds were identified by SAFE. Among them, 2-methylpyrazine, 2,6-dimethylpyrazine, dihydroactinidiolide, coumarin, and γ-butyrolactone were only recovered by SAFE; 2-methylbutanal, 3-methylbutanal, isophorone, α-ionone, β-ionone, β-cyclocitral, 2-phenyl-2-butanal, myrtenal, cis-jasmone, dimethyl disulfide, dimethyl sulfide, and 2-methylpropanal were only recovered by HS-SPME. The results of this experiment also verified the previous conclusions: SAFE is effective in extraction of pyrazine and coumarin derivatives, while HS-SPME has a better effect on aldehydes and ketones [17]. Because the TICs of HS-SPME and SAEF are quite different (Figure S1), better results can be obtained when they are used in combination.

#### 2.1.2. Quantitation of Aroma Compounds

According to the above experimental conclusions, the key aroma compounds were quantitatively analyzed by the standard curve (Table 2). The relevant information is supplemented in Table S1.

These compounds are divided into nine chemical categories: alcohols, aldehydes, acids, esters (lactones), ketones, hydrocarbons, sulfide, pyrazines, and others. Alcohol is a very important chemical category in black tea based on the composition and concentration of the compounds. The quantitative results showed that phenethyl alcohol, linalool, geraniol, and benzyl alcohol have relatively high concentrations in different kinds of black tea. As far as we know, this is the first report on α-methylbenzyl alcohol in black tea, and α-methylbenzyl alcohol (cas:98-85-1) is the isomer of β-phenethyl alcohol (cas:60-12-8). Its aroma is different from β-phenethyl alcohol, i.e., α-methylbenzyl alcohol is a lilac-like aroma, while phenylethyl alcohol is a warm rose aroma.

It is worth noting that under the same detection conditions, some compounds were only detected in a certain sample and the concentrations are relatively high, which is probably an important reason for the differences in aromas of different black teas. 4-Methoxybenzaldehyde (>20 μg/L) was only identified in DH; 2-pentanol (>100 μg/L), 1-penten-3-ol (>200 μg/L), 2-heptanone (>20 μg/L), methyl hexanoate (>50 μg/L), and isophorone (>50 μg/L) were only identified in KM; 2,6-dimethylpyrazine (>50 μg/L) was only identified in JJM.

#### 2.1.3. Odor Activity Values (OAVs)

Christian Schuh and Peter Schieberle have stated that AEDA cannot fully explain the aroma contribution of a compound, and the odor activity value still needs to be introduced [21,22]. The OAVs were calculated based on the concentration obtained by the standard curve and the threshold value of the compound in water; an OAV >1 was considered to be an important aroma compound. Threshold determinations were performed on compounds involved in aroma synergy studies, and other threshold data were obtained from the literature. 

Finally, the OAVs of 52 compounds were calculated. The compound with the highest OAV in JJM is linalool (242.34), the compound with the highest OAV in KM is hexanal (537.30), and the compound with the highest OAV in DH is furfuryl alcohol (239.89). In addition, the OAVs of the following compounds showed a high degree of aroma contribution: geraniol, phenethyl alcohol, (*Z*)-3-hexenol, phenylacetaldehyde, furfural, 2-methylbutanal, 3-methylbutanal, 3-methylnonane-2,4-dione, β-damascenone, methyl salicylate, 2-heptanone, β-myrcene, dimethyl sulfide, and 2,6-dimethylpyrazine. The OAVs of these compounds were greater than 10. There were 39 compounds with OAV greater than 1.

#### 2.1.4. The Differences among the Three Kinds of Black Tea

**Figure 1:**
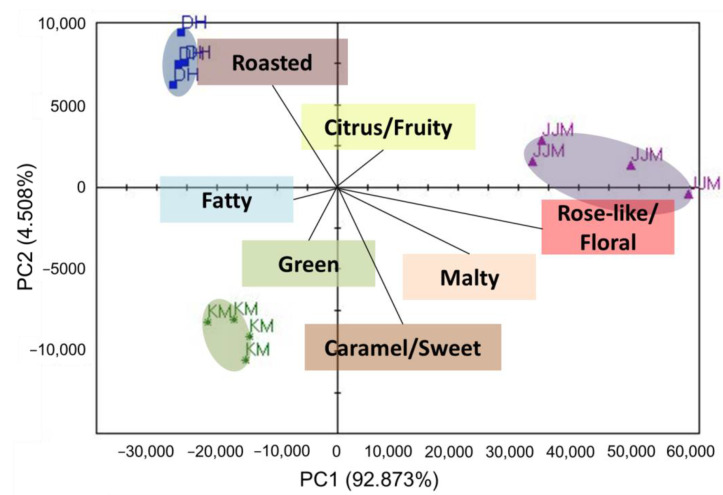
Principal component analysis and sensory analysis of three black teas.

According to the GC-MS results, there were significant differences in the concentrations of the compounds in the three tea samples. On this basis, the ultra-fast gas chromatography electronic nose was performed to show the differences among the samples. The response values (three samples, repeated four times) of the HERACLES II electronic nose of different samples were subjected to principal component analysis (PCA), and the results are shown in Figure 1. The PCA results show that the differences are very obvious, mainly concentrated on the principal component 1 (92.873%), and the validation score reaches 92.

The sensory analysis of the three tea infusions with the previously determined black tea aroma attributes, and the sensory results and the PCA results were combined as shown in Figure 1. The distance between the descriptor and the different samples in the figure reflect the preference, the higher the score of the aroma note, the shorter the distance, and vice versa.

The sensory results corresponded to the previous OAV values. JJM had the highest score for floral fragrance, and linalool (citrus, floral) had the highest OAV; KM had the highest score for green, and hexanal (grass) had the highest OAV; DH had the highest score for roasted, and furfuryl alcohol (caramel, sweet) had the highest OAV.

### 2.2. Perceptual Interactions

At present, many studies have proven that mixing different compounds will affect their aroma perception [23]. However, reports on the synergistic effects of flavor compounds in black tea are scarce. As far as we know, in recent years, only Zhu et al. studied the synergistic effect of 3-methylbutanal and 2-methylbutanal in oolong tea based on their similar structure [24]. However, there is no research on the interaction of the common key aroma compounds in black tea.

#### 2.2.1. Selection of Aroma Compounds

Black tea is a fully fermented tea, and its floral aroma plays an important role in the complex aroma [25]. As compared to other teas, black tea has a stronger, sweeter floral aroma. Therefore, this study focused on the effect of aroma perception of key aroma ester (MeSA) with floral compounds.

This part took the floral compounds as the research object, and common floral compounds in different black teas were used as targets for sensory interaction, specifically including linalool, phenylethyl alcohol, benzyl alcohol, geraniol, nerol, alpha-terpineol, cis-jasmone, β-ionone, β-ocimene, and benzeneacetaldehyde.

Another target compound is MeSA. Most importantly, because it was regarded as an important key aroma compound in various black teas; secondly, among the esters detected in this study, it had a high concentration (0.28–1.51 mg/kg), also a high odor activity value (OAV > 3) and FD (256–512); and its aroma quality similar to wintergreen oil was also very important. Therefore, MeSA and floral compounds were selected for the perception interaction, in order to achieve the purpose of providing theoretical guidance for most black teas.

#### 2.2.2. Interactions among MeSA and Floral Compounds

In order to obtain a better understanding of the interactions of MeSA on the floral compounds of tea infusion, the effect of MeSA on each floral compound was explained by comparing the measured threshold with the theoretical value calculated by the Feller additive model; the fitted sigmoid curve was used to evaluate the aroma interaction during binary mixing [22,26,27]. The binary mixture was prepared according to the average concentration ratio of MeSA and floral compounds actually detected in the tea infusion.

**Figure 2:**
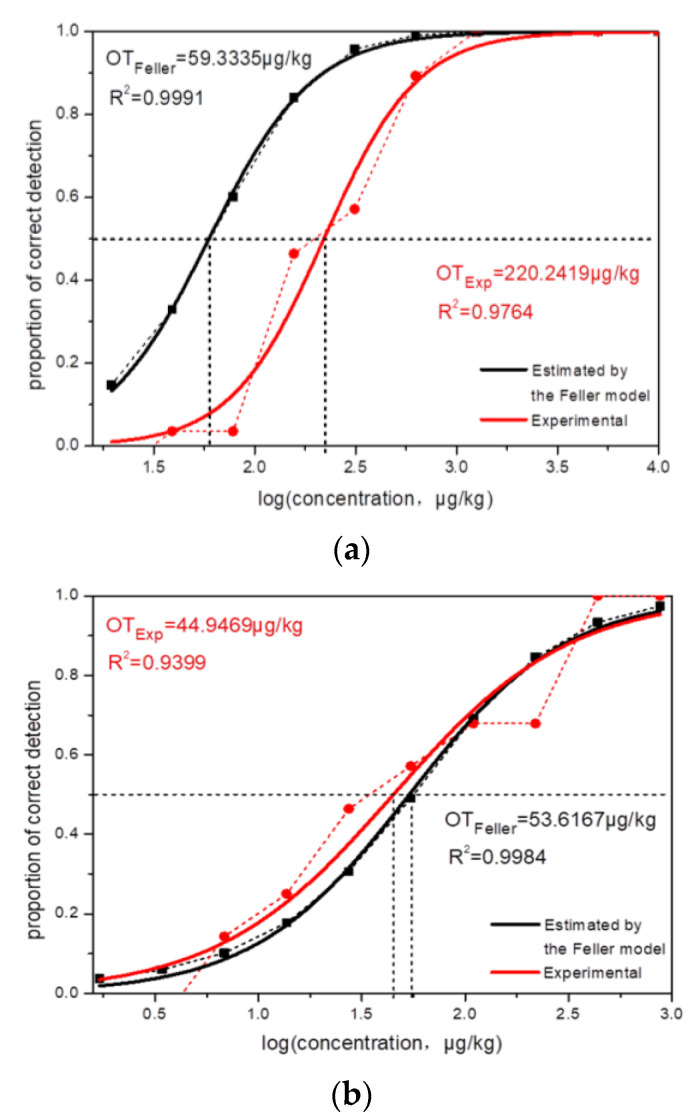

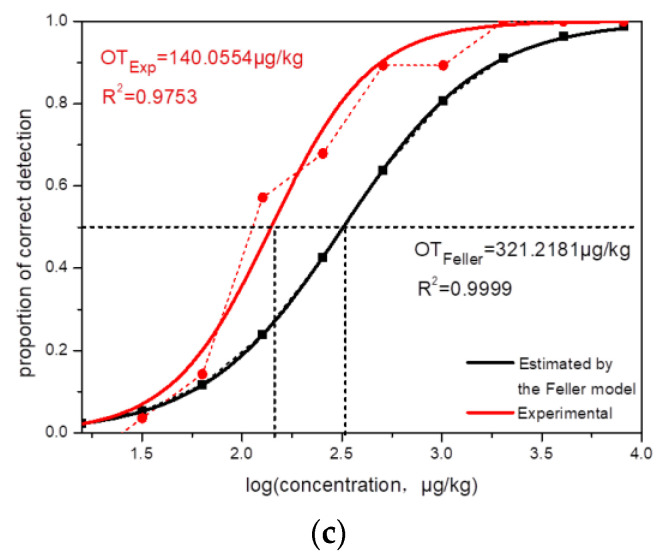
Interactions between methyl salicylate and floral compounds: (**a**) MeSA + phenylacetaldehyde, masking effect, (**b**) MeSA + cis-jasmone, addition Effect. (**c**) MeSA + benzyl alcohol, synergistic effect.

Among the 10 groups of binary mixtures tested in this study, five groups showed masking effects, four groups showed synergistic effects, and one group showed additive effects. Among them, the actual threshold was greater than the theoretical threshold obtained by Feller’s additive model: for masking effect, including MeSA/linalool (R = 5.8587), MeSA/phenylacetaldehyde (R = 3.7119), MeSA/geraniol (R = 5.4815), MeSA/phenethyl alcohol (R = 2.7309), MeSA/β-ionone (R = 2.6626); the ratio of the actual threshold to the theoretical threshold was between 0.5 and 1 for addition effect, MeSA/cis-jasmone (R = 0.8140); the ratio of the actual threshold to the theoretical threshold was less than 0.5 for synergistic effect, MeSA/nerol (R = 0.4369), MeSA/benzyl alcohol (R = 0.4360), MeSA/α-terpineol (R = 0.4736), MeSA/β-ocimene (R = 0.4953). Part of the results are shown in Figure 2.

Although most of the previous studies have been based on the same or different chemical structures, the chemical complexity of compounds would affect the perception of interaction [28,29]. Experimental results proved that although the chemical categories of the mixture may be the same, the interaction effects were not exactly identical.

#### 2.2.3. Electronic Nose Response to the Interaction of Aroma Perception

The above experiment proved that MeSA did have a very important effect on the floral fragrance of tea. In order to observe its effect on tea fragrance, MeSA (0.5, 1, 5, and 10 μL), tea infusion, tea infusion, and MeSA (0.5, 1, 5, and 10 μL) of the mixture were set up and tested for the HERACLES electronic nose. JJM was selected as the tea sample for the experiment.

**Figure 3:**
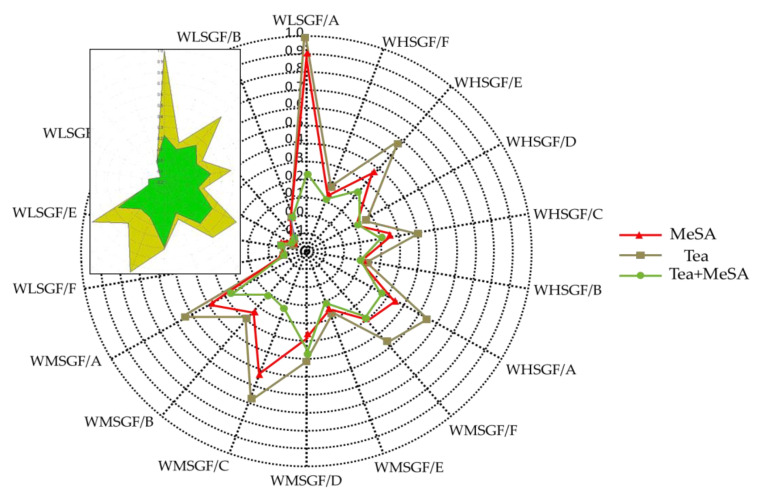
The expression of the interaction of aroma perception on the electronic nose.

The results were unexpected: The addition of MeSA had a significant effect on the overall aroma. The masking effect of different concentrations of MeSA on tea aroma was confirmed. For a clearer and more intuitive representation, the electronic nose aroma profile of three samples of 5 μL MeSA, tea infusion, tea infusion and 5 μL MeSA mixture are shown (Figure 3).

In order to highlight the effect, the small window showed the tea infusion and the mixture of tea infusion and MeSA. There was no doubt that the addition of MeSA reduced the response value of all sensors, which means that MeSA masked the floral in the tea, and also other aroma attributes.

The electronic nose confirmed the masking effect of MeSA on the floral aroma of black tea. Combined with our analysis of the synergy/masking/additive effect of our binary mixture, the possible reasonable explanation was that MeSA and occupying higher concentrations of compounds such as linalool, phenethyl alcohol, and geraniol showed masking effects. The concentration of the floral compounds that had a synergistic effect with it was relatively low. Therefore, in summary, MeSA can achieve the effect of masking floral fragrance in black tea.

#### 2.2.4. The Correlation between Steven’s Law and Perceptual Interactions

Steven’s law is expressed as:

I = kC^n^ (1)

where I is the intensity of the aroma and C is the concentration of the compound. 

V.V. Kamadia once applied it to aroma perception [30]. In this study, these 11 compounds were tested individually. There were 10 concentration points for each compound, corresponding to 10 aroma intensity values. According to the Steven function, the optimal k and n values were obtained through constant iteration, and the fit of this nonlinear regression was all above. 900, as shown in the Table 3.

Among them, n > 1 was considered to be expansiveness and n < 1 was considered to be compression, which means that the odor concentration increases more rapidly or slowly as the concentration increases [31]. Therefore, we selected n as the key research object and connected it with the effect results obtained by the S curve. We analyzed the differences in their n values in the binary combination, and found that when the difference of n was greater than 0.3, it meant that the aroma intensity of these two compounds changed greatly with an increase in concentration, which was expressed as a masking effect; when the difference of n was between 0.2 and 0.3, it meant that there was a genera change in the aroma intensity of these two compounds with an increase in the concentration, which was expressed as an additive effect; when the difference of n was less than 0.2, it meant that there was a slight change in the aroma intensity of these two compounds with an increase in the concentration, and it was expressed as a synergistic effect. As shown in Table 3.

To further verify this point, we mixed MeSA with floral compounds to obtain a binary mixture, and then regarded the mixture as a whole and calculated its value of n, which was denoted as N here. When a synergistic effect was shown, it means that after mixing, the release of aroma could be further promoted. Therefore, N must be greater than the average of n_a_ and n_b_ at this time. In the same way, N must be less than the average of n_a_ and n_b_ when a masking effect was shown, as shown in Table 3.

Logically, the n value can represent the release speed of the aroma to a certain extent, and the R value obtained by Feller’s additive model can also reflect the degree of interaction. We tried to fit it and found that when the binary compounds were mixed, the n difference had a good exponential relationship with R, and R^2^ reached 0.914. The fitted formula is:

R = 0.277e^6.986x^. (2)

It expects to connect the Steven’s law to Feller’s additive model and reach a general direction that can predict the interaction of aroma. However, it should be stated that this is only summarized by this research, and is not based on a huge amount of experimental data.

## 3. Material and Methods

### 3.1. Materials

Three representative black teas from China were selected for this research (Jinjunmei (JJM), Keemun (KM), and Yunnan Dianhong (DH)). Specifically, JJM was purchased from Zhengshan Tea Industry Co., Ltd., Wuyishan National Nature Reserve, Fujian, with a production date of 15 May 2020; KM was purchased from Anhui Keemun Black Tea Development Co., Ltd., and was produced on 18 September 2020; DH was purchased from Kunming Dianpinhao Tea Co., Ltd., Yunnan Province, with a production date of 27 September 2020. All tea samples come from their specialty areas.

### 3.2. Chemicals

All reference compounds were purchased from commercial sources.

Experimental distilled water was purchased from Watson Water Company, Inc. (Guangzhou, China). Dimethyl sulfide (97%), 2-methylbutanal (≥97%), 3-methylbutanal (≥97%), 2-ethylfuran (≥97%), pentanal (≥97%), hexenal (≥97%), dimethyl disulfide (≥97%), 1-penten-3-ol (≥97%), β-myrcene (≥97%), 2-heptanone (≥97%), methyl hexanoate (≥97%), D-limonene (≥97%), (E)-2-hexenal (≥97%), 2-pentylfuran (≥97%), β-ocimene (≥97%), styrene (≥97%), 2-methylpyrazine (≥97%), 2-pentenol (≥97%), 2,6-dimethylpyrazine (≥97%), 2-ethylprazine (≥97%), 6-methyl-5-hepten-2-one (≥97%), 1-hexanol (≥97%), (Z)-3-hexen-1-ol (≥97%), 1-octen-3-ol (≥97%), furfural (≥97%), cis/trans-linaloloxide (≥97%), 2-ethyl-1-hexanol (≥97%), (E, E)-2,4-heptadienal (≥97%), benzaldehyde (≥97%), linalool (≥97%), longifolene (≥97%), (E,E)-3,5-octadien-2-one (≥97%), isophorone (≥97%), 5-methylfurfural (≥97%), 4-terpinenol (97%), γ-butyrolactone (97%), hotrienol (97%), 1-ethyl-1H-pyrrole-2-carbaldehyde (≥97%), beta-cyclocitral (≥97%), (1R)-(-)-myrtenal (≥97%), benzeneacetaldehyde (≥97%), furfuryl alcohol (≥97%), neral (≥97%), α-terpineol (≥97%), benzyl acetate (≥97%), naphthalene (≥97%), methyl salicylate (≥97%), nerol (≥97%), hexanoic acid (≥97%), α-methylbenzyl alcohol (≥97%), geraniol (≥97%), α-ionone (≥97%), β-ionone (≥97%), benzyl alcohol (≥97%), phenylethyl alcohol (≥97%), (E)-3-hexenoic acid (≥97%), (E)-2-hexenoic acid (≥97%), 2-acetyl pyrrole (≥97%), β-damascenone (≥97%), 2-phenyl-2-butenal (≥97%), cis-jasmone (≥97%), 2-formyl-1H-pyrrole (≥97%), 3-methylnonane-2,4-dione (≥97%), 4-methoxybenzaldehyde (≥97%), geranic acid (≥97%), dihydroactinidiolide (≥97%), benzoic acid (≥97%), coumarin (≥97%) were purchased from Alfa Aesar Corporation (Tianjin, China). Alkanes in solution (C5–C30) and internal standard 1,3-dichlorobenzene were purchased from ANPEL Laboratory Technologies Inc. (Shanghai, China).

### 3.3. Tea Infusion Preparation

#### 3.3.1. Solvent-Assisted Flavor Evaporation (SAFE)

Hot distilled water (90 °C, 150 mL) was added to the black tea (3 g) in a conical flask. After equilibration for 3 min, it was filtered to obtain the tea infusion. Then 40 μL of 1,3-dichlorobenzene (220 mg/kg in EtOH) was added to tea infusion as an internal standard. To extract the aroma more effectively, we used a SAFE apparatus with a vacuum pump, and ensured that the temperature of circulating water and water bath were both 40 °C and the pressure was 10^−4^ pa to distill the tea infusion in the whole experiment. After the liquid collected by SAFE was naturally thawed, it was extracted three times with 50 mL dichloromethane, then, the extracts were combined and dried overnight with anhydrous Na_2_SO_4_ [4]. Finally, the extract was concentrated to 1 mL under a nitrogen stream.

#### 3.3.2. Headspace Solid-Phase Microextraction (HS-SPME)

The method was based on the literature and some optimizations as follows: Take 0.2 g of ground tea leaves, add 10 mL of hot distilled water (90 °C), and add 0.2 g of sodium chloride in a 15 mL headspace bottle, equilibrated for 3 min, and supplement with 5 μL of 1,3-dichlorobenzene (21 mg/kg in EtOH) as an internal standard [4]. The headspace SPME fiber (Supelco, Inc., Bellefonte, PA, USA) was 50/30 μm DVB/CAR/PDMS. Heating and extracting were performed in a 50 °C water bath for 40 min.

### 3.4. Gas Chromatography-Mass Spectrometry (GC-MS): Compound Identification and Quantification

An Agilent 7890 gas chromatograph (GC) system coupled with a 5973C mass spectrometer (MS) was used to analyze the aroma compounds of black tea. The operated mode was electron ionization (EI) mode (70 eV, ion source temperature 230 °C), and the quadrupole was in scanning mode (scanning range *m*/*z* 30–450 and scanning rate 1 scan/sec). With helium (purity = 99.999%) as the carrier gas (the flow velocity was 1.8 mL/min), two columns with different polarities were used to separate the compounds: Innowax-Wax (60 m × 0.25 mm i.d. × 0.25 μm film thickness, Agilent Technologies) and DB-5 (60 m × 0.25 mm i.d. × 0.25 μm film thickness, Agilent Technologies). The oven temperature was optimized: Initial oven temperature was 40 °C, holding for 6 min, then, ramped to 150 °C by 3 °C/min, and then, increased to 230 °C at a rate of 6 °C/min with a 15 min hold [32].

The compounds were identified by comparing with the mass spectra in the NIST17 library, comparing the RIs with the previous reported in the literature, or comparing the mass spectra and RI with the reference chemical. The aroma active compounds were quantified by constructed external standard curves. The slope and intercept were fitted by the ratio of the peak area of the authentic standard to the internal standard and the ratio of the authentic standard’s concentration to the concentration of the internal standard (refer to the previous research description [33,34,35]).

### 3.5. Gas Chromatography-Olfactometry (GC-O) and Aroma Extract Dilution Analysis (AEDA)

The GC-MS system (Agilent 7890, Agilent Technologies, Santa Clara, CA, USA) was connected to the Olfactory Evaluation Port (ODP-3, Gerstel, Mullheim an der Ruhr, Germany) for the aroma analysis. After the aroma substances were separated by the chromatographic column, they were sent to the MS detector and olfactory evaluation port at a ratio of 1:1. The column and oven temperature program parameters were the same as GC-MS. Ionization was at 70 eV and the scan range was 30–450 *m*/*z*.

For the samples processed by HS-SPME, the perceived aroma description and aroma intensity (AI) were recorded. The AI scale was from 0 (nil) to 10 (extreme). For the samples processed by SAFE, they were gradually diluted with dichloromethane as the solvent, and the highest dilution was 1:512 [36]. The maximum dilution of each aroma compound was determined as the flavor dilution (FD) of this compound.

### 3.6. Sensory Analysis

#### 3.6.1. Sensory Panel

This study was reviewed and approved by the Shanghai Institute of Technology and informed consent was obtained from each subject prior to their participation in the study. In order to ensure the reliability of the results of all sensory experiments, a total of 45 subjects (23 males and 22 females, with an average age of 25) participated in the screening of the sensory group. The subjects came from the School of Perfume and Aroma Technology of Shanghai Institute of Technology (Shanghai, China). The test was carried out in the sensory evaluation room. Sensory panel 1 consisted of 10 subjects (5 males and 5 females, with an average age of 24) and was responsible for the aroma intensity and AEDA analysis during the GC-O analysis. Each experiment was repeated three times for each subject, and the average value of the aroma intensity score was taken as the final result. Sensory panel 2 consisted of 14 subjects (7 males and 7 females, with an average age of 25) and was responsible for threshold determination.

The subjects had all received professional training, and could sensitively identify the different aroma qualities in black tea. In addition, everyone on the panel completed 20 specific trainings with tea infusions, 45 min per training.

#### 3.6.2. Odor Threshold Determination

The aroma compounds to be tested were dissolved in water at the initial concentration and serially diluted to obtain 10 samples with successively decreasing concentrations, which were subjected to mandatory screening according to the three-alternative forced choice (3-AFC) methodology [37]. Each concentration was presented along with two water samples per level. Then, the threshold was calculated according to the reported method [38].

#### 3.6.3. Exploration of the Interaction of Aroma Perception

The 10 concentration gradients of each group of binary mixtures were sniffed from high to low. Each concentration sample was equipped with two bottles of blank solution. As previously reported, the number of people who can identify correctly were recorede, and fit to the Feller’s additive model with the corrected sniffing probability [22,39].

#### 3.6.4. Quantitative Descriptive Analysis (QDA)

QDA was performed by the second sensory team to evaluate three different black tea samples [40]. After discussing the aroma characteristics of black tea infusion, the assessors determined the seven aroma basic attributes: roasted note, caramel/sweet note, rose-like/floral note, green note, citrus/fruity note, fatty note, and malty note.

### 3.7. Odor Activity Values (OAVs)

The OAV of a compound was calculated by dividing the calculated concentrations with sensory thresholds (in water), which were obtained from the literature or detected in this study.

### 3.8. Electronic Nose Analysis (ENA)

The aroma profile description of the ultra-fast gas chromatography electronic nose (HERACLES ll, Alpha M.O.S., Toulouse, France) was used to analyze the composition difference of the three black teas. The samples were prepared according to the previous tea/water ratio and 3 mL was placed in each 15 mL headspace vial. The injection volume was 5000 μL. Each sample was incubated at 50 °C for 20 min under agitation (500 rpm) and the injection speed was 125 μL/s.

An electronic (HERACLES, Alpha M.O.S., Toulouse, France) nose with 18 metal oxide sensors was used to verify the enhancement/decrease effect of MeSA on each compound with floral aroma and the overall floral fragrance. The experimental parameters were the same as the HERACLES ll electronic nose. All electronic nose analyses were repeated 5 times.

### 3.9. Statistical Analysis

Analysis of variance was performed using the Duncan test for identifying significant variance, with significance indicated by *p* ≤ 0.05 and the establishment of a regression model. The analysis was completed with the SPSS v26.0 software (SPSS, Chicago, IL, USA).

The principal component analysis used the electronic nose-related software Alphasoft V12.44 (Alpha M.O.S., Toulouse, France).

The Feller’s additive model was employed as modeled in the Origin 9.0 software (OriginLab Corporation, Northampton, MA, USA).

## 4. Conclusions

This study focused on three kinds of black tea samples: Jinjunmei, Keemun, and Dianhong and analyzed their aroma characteristics; 70 compounds including α-methylbenzyl alcohol (isomer of β-phenylethanol) were identified as odorants. There were 39 different compounds that made important contributions to the aroma of black tea (OAV > 1), which enriched the database of aroma compounds of black tea. In addition, the interaction between MeSA and floral compounds in tea was explored through the Feller’s additive model, and the results showed that when the concentration of MeSA in tea was increased, the release of floral was depression. On this basis, the change of the parameter n in Steven’s law during binary mixing was very attractive and, in this study, we found that it had an exponential relationship with the high degree of fit of the R value. However, it is worth stating that this relationship was only a conclusion based on the limited data of this study. Next, we will further study the effects of other aroma compounds commonly found in different black teas on the overall floral fragrance. This research has enriched the theoretical knowledge related to the aroma of black tea, and we hope it provides help for simulating the natural aroma of black tea.

## Figures and Tables

**Table 1:** Comparison of flavor dilution factor (FD) and aroma intensity (AI) of the aroma compounds identified in Jinjunmei (JJM), Keemun (KM), and Dianhong (DH) black teas.

No	Aroma Compounds	LRI ^a^		Odor Quality ^d^	JJM		KM		DH		ID ^e^
		DB-5 ^b^	HP-Innowax ^c^		AI	FD	AI	FD	AI	FD	
1	Dimethyl sulfide	<600	750	Corn	0.5	-	4.9	-	1.2	-	RI, MS, A
2	2-Methylpropanal	<600	810	Fresh aldehydic	2.1	-	3.7	-	3.0	-	RI, MS, A
3	2-Methylbutanal	641	909	Musty, cocoa	3.2	-	2.6	-	1.9	-	RI, MS, A
4	3-Methylbutanal	653	913	Fruity, chocolate	2.5	-	3.1	-	2.2	-	RI, MS, A
5	2-Ethylfuran	717	950	Bread, sweet	1.2	32	1.9	32	6.7	64	RI, MS, A
6	Pentanal	694	975	Fermented, bready	2.7	16	2.4	32	-	-	RI, MS, A
7	Hexanal	803	1077	Fresh green grass	3.8	32	4.5	64	4.1	32	RI, MS, A
8	Dimethyl disulfide	775	1081	Sulfurous, vegetable	-	-	-	-	3.5	-	RI, MS, A
9	1-Penten-3-ol	686	1156	Green, radish	-	-	2.1	32	-	-	RI, MS, A
10	β-Myrcene	999	1165	Peppery, woody	3.1	32	1.9	16	2.6	16	RI, MS, A
11	2-Heptanone	898	1178	Fruity, cheese	-	-	2.1	8	-	-	RI, MS, A
12	Methyl hexanoate	925	1181	Fruity, pineapple	-	-	2.4	16	-	-	RI, MS, A
13	d-limonene	-	1189	Citrus, orange	1.9	32	1.2	32	1.8	32	RI, MS, A
14	(*E*)-2-Hexenal	848	1219	Green, fruity	4.2	64	5.3	128	3.9	32	RI, MS, A
15	2-Pentylfuran	994	1226	Fruity, green	3.1	32	3.6	64	2.1	32	RI, MS, A
16	β-Ocimene	1030	1249	Citrus, tropical	2.5	16	2.6	16	3.4	16	RI, MS, A
17	Styrene	898	1256	Sweet, floral	0.3	4	0.6	8	0.5	4	RI, MS, A
18	2-Methylpyrazine	834	1264	Popcorn, nutty	-	64	-	32	-	32	RI, MS, A
19	2-Pentenol	765	1324	Fruity	-	-	3.8	16	-	-	RI, MS, A
20	2,6-dimethylpyrazine	902	1335	Roasted, coffee	-	16	-	-	-	-	RI, MS, A
21	2-Ethyl-pyrazine	914	1337	Nutty, musty	3.2	64	-	-	2.9	64	RI, MS, A
22	6-Methyl-5-hepten-2-one	990	1336	Citrus, green	2.9	64	3.5	128	1.7	32	RI, MS, A
23	1-Hexanol	854	1346	Oily, fruity	3.4	16	2.1	32	1.9	16	RI, MS, A
24	(*Z*)-3-Hexen-1-ol	863	1378	Fresh, grass	4.1	256	4.1	256	4.8	512	RI, MS, A
25	1-Octen-3-ol	976	1444	Mushroom, earthy	-	-	4.7	128	6.8	256	RI, MS, A
26	Furfural	831	1465	Sweet, woody	6.8	128	6.4	256	6.1	128	RI, MS, A
27	cis/trans-Linaloloxide	1090/1079	1468/1438	Citrus, floral	5.9	128	4.5	128	6.9	128	RI, MS, A
28	2-ethyl-1-hexanol	1036	1481	Citrus, sweet	2.5	16	2.4	16	2.1	32	RI, MS, A
29	(E,E)-2,4-Heptadienal	1022	1494	Fatty, green	5.7	64	2.2	64	-	-	RI, MS, A
30	Benzaldehyde	965	1527	Almond, oily	3.5	32	5.4	64	5.3	32	RI, MS, A
31	Linalool	1112	1541	Citrus, floral	6.1	32	6.8	64	7.9	128	RI, MS, A
32	Longifolene	1408	1546	Sweet, woody	1.1	8	-	-	2.8	16	RI, MS, A
33	(E,E)-3,5-Octadien-2-one	1098	1562	Fruity, green	4.2	64	4.6	128	-		RI, MS, A
34	Isophorone	-	1570	Sweet, woody	-	-	2.8	-	-	-	RI, MS, A
35	5-methyl furfural	982	1575	Sweet, maple	5.6	64	4.7	32	5.1	64	RI, MS, A
36	4-Terpinenol	1169	1599	Pepper woody	2.1	16	-	-	4.1	32	RI, MS, A
37	gamma-Butyrolactone	911	1604	Creamy, oily	-	16	-	32	-	8	RI, MS, A
38	Butanoic acid	920	1609	Sharp acetic	-	-	1.9	16	-	-	RI, MS, A
39	Hotrienol	1119	1614	Lavender	2.4	8	1.1	4	1.9	8	RI, MS, A
40	1-Ethyl-1H-pyrrole-2-carbaldehyde	1029	1618	Burnt, roasted, smoky	5.2	128	6.9	256	4.9	64	RI, MS, A
41	beta-Cyclocitral	1241	1625	Tropical saffron, herbal	4.1	-	5.8	-	-	-	RI, MS, A
42	(1R)-(-)-Myrtenal	-	1634	Sweet, minty	3.6	-	-	-	-	-	RI, MS, A
43	Benzeneacetaldehyde	1048	1654	Floral, honey	3.9	256	8.1	128	4.9	128	RI, MS, A
44	Furfuryl alcohol	1075	1665	Sweet, caramel	4.7	64	-	-	3.5	32	RI, MS, A
45	3-methylnonane-2,4-dione	1445	1680	Hay like	-	-	5.5	64	3.6	16	RI, MS, A
46	Neral	1247	1684	Lemon peel, citrus	4.6	64	5.3	64	-	-	RI, MS, A
47	alpha-Terpineol	1199	1693	Lilac, woody,	2.2	16	1.8	8	1.8	8	RI, MS, A
48	Benzyl acetate	1164	1732	Fruity, floral	3.9	16	4.2	32	-	-	RI, MS, A
49	Naphthalene	1209	1751	Balmy	0.8	8	1.1	16	0.9	8	RI, MS, A
50	Methyl salicylate	1204	1785	Wintergreen	5.7	512	6.2	256	8.7	512	RI, MS, A
51	Nerol	1230	1793	Neroli, citrus	3.9	64	4.4	128	-	-	RI, MS, A
52	Hexanoic acid	986	1802	Fatty, cheesy	2.2	32	1.9	32	1.7	32	RI, MS, A
53	β-Damascenone	1386	1822	Floral, fatty	-	-	4.5	16	-	-	RI, MS, A
54	α-Methylbenzyl alcohol	1051	1827	Fresh sweet	4.6	64	2.1	32	-	-	RI, MS, A
55	Geraniol	1235	1841	Sweet, floral	5.1	256	6.1	512	3.8	128	RI, MS, A
56	α-Ionone	1439	1858	Woody	-	-	2.1	-	-	-	RI, MS, A
57	Benzyl alcohol	1030	1876	Floral	2.9	64	4.5	64	3.1	32	RI, MS, A
58	Phenylethyl alcohol	1110	1912	Rose, floral	5.2	128	5.5	256	6.5	512	RI, MS, A
59	(E)-3-hexenoic acid	1019	1914	Green, fruity	3.6	64	4.3	128	3.8	128	RI, MS, A
60	(E)-2-hexenoic acid	-	1917	Fruity, sweet	3.3	64	4.1	128	3.6	128	RI, MS, A
61	2-Acetyl pyrrole	1065	1928	Musty, sweet	1.5	16	3.2	64	2.8	64	RI, MS, A
62	2-Phenyl-2-Butenal	1281	1941	Floral, black tea	3.2	-	2.9	-	4.7	-	RI, MS, A
63	β-Ionone	1494	1947	Woody, floral	3.6	-	3.4	-	3.7	-	RI, MS, A
64	cis-Jasmone	1391	1955	Woody, herbal	3.9	-	4.5	-	-	-	RI, MS, A
65	2-Formyl-1H-pyrrole	1015	1983	Musty, beefy	3.1	32	3.5	64	2.2	16	RI, MS, A
66	4-Methoxybenzaldehyde	1251	2004	Sweet, warm	-	-	-	-	1.9	16	RI, MS, A
67	Geranic acid	1349	2332	Green, woody	1.1	32	0.6	8	0.7	8	RI, MS, A
68	Dihydroactinidiolide	1495	2349	Musk	-	64	-	32	-	-	RI, MS, A
69	Benzoic acid	1191	2442	Faint, balsam	0.5	4	0.7	8	3.5	16	RI, MS, A
70	Coumarin	1435	2468	Sweet	-	32	-	16	-	-	RI, MS, A

^a^ LRI, linear retention index; ^b^ DB-5, non-polar GC Column; ^c^ HP-Innowax, polar GC Column; ^d^ Odor quality perceived by GC-O analysis; ^e^ identification method, aroma(A), retention indices (RI) and mass spectra (MS) agree with the authentic compounds.

**Table 2:** Concentrations, odor thresholds and odor activity values (OAVs) of key aroma compounds in Jinjunmei (JJM), Keemun (KM), and Dianhong (DH) black teas.

No.	Odorant	Identification	Standard Curve			Concentration (μg/kg)						Content Range(%)	OTs ^a^ (μg/L)	OAV		
			Slope	Intercept	R^2^	JJM	RSD (%)	KM	RSD (%)	DH	RSD (%)			JJM	KM	DH
Alcohols																
1	Linalool	MS, RI, Std	0.9262	0.0178	0.9957	4604.46a ^b^	1.62	543.13c	8.47	2084.49b	3.83	4.72–10.38	19 *	242.34	28.59	109.71
2	Geraniol	MS, RI, Std	0.0163	−0.4096	0.9963	474.37b	0.57	6185.74a	3.34	624.75b	1.59	0.86–8.80	27 *	17.57	229.10	23.14
3	Phenylethyl alcohol	MS, RI, Std	0.9769	0.8083	0.9992	21066.85a	9.74	4235.09b	9.07	1465.43c	9.18	4.88–10.81	772 *	27.29	5.49	1.90
4	(Z)-3-Hexenol	MS, RI, Std	1.0889	0.0168	0.9924	2177.45a	4.53	347.50b	8.72	233.43b	2.77	0.39–1.14	70	31.11	4.96	3.33
5	Benzyl alcohol	MS, RI, Std	1.0010	0.2018	0.9894	14261.10a	9.13	10245.28b	6.83	251.02c	2.86	2.06–6.59	11,076 *	1.29	0.92	0.02
6	*cis* -Linaloloxide	MS, RI, Std	0.6374	0.0864	0.9837	172.04c	8.35	508.25a	9.05	348.06b	3.78	2.04–5.59	320	0.54	1.59	1.09
7	Nerol	MS, RI, Std	0.0185	0.0068	0.9742	593.96	2.76	254.35	6.53	-^c^	-	0.69–1.06	528 *	1.12	0.48	-
8	α-Terpineol	MS, RI, Std	0.1873	−0.1682	0.9194	49.29a	3.14	35.16b	2.01	22.10c	4.40	0.34–0.59	404 *	0.12	0.09	0.05
9	1-Hexanol	MS, RI, Std	0.1210	−0.0421	0.9817	17.58b	5.38	20.30a	5.73	19.71ab	7.49	0.23–0.41	500	0.04	0.04	0.04
10	*trans*-Linaloloxide	MS, RI, Std	0.9345	0.0458	0.9643	172.99b	5.78	234.20a	2.89	72.54c	3.58	0.86–2.58	320	0.54	0.73	0.23
11	α-Methylbenzyl alcohol	MS, RI, Std	1.0985	0.0035	0.8995	64.67	7.82	23.24	5.00	-	-	0.05–0.07	-	-	-	-
12	2-Pentenol	MS, RI, Std	0.9921	0.0128	0.9005	-	-	191.81	8.91	-	-	0.25–0.30	400	-	0.48	-
13	1-Penten-3-ol	MS, RI, Std	0.6072	0.0415	0.9219	-	-	298.20	9.43	-	-	0.4–0.45	400	-	0.75	-
14	4-Terpinenol	MS, RI, Std	0.2376	−0.2192	0.9575	16.92	0.54	-	-	16.38	1.63	0.07–0.08	-	-	-	-
15	2-Ethyl-1-hexanol	MS, RI, Std	1.1905	0.0465	0.9613	241.52a	9.27	77.50b	4.02	257.13a	6.26	0.31–0.77	300	0.81	0.26	0.86
16	Furfuryl alcohol	MS, RI, Std	1.1681	0.0609	0.9894	251.82	6.54	-	-	1079.49	6.52	0.50–1.18	4.5	55.96	-	239.89
17	1-Octen-3-ol	MS, RI, Std	0.0389	0.0128	0.9952	-	-	94.07	4.60	14.21	1.14	0.18–0.55	45	-	2.09	0.32
18	Hotrienol	MS, RI, Std	0.6532	0.0656	0.9738	47.88c	3.86	233.60a	5.26	148.76b	3.95	0.57–2.57	110	0.44	2.12	1.35
Aldehydes																
1	(E)-2-Hexenal	MS, RI, Std	0.7946	0.0345	0.9952	261.79a	7.98	164.93b	8.50	177.96b	4.08	0.16–0.34	82	3.19	2.01	2.17
2	Benzeneacetaldehyde	MS, RI, Std	0.7957	0.1310	0.9917	143.75c	2.89	1623.40a	7.15	461.80b	7.21	0.78–2.54	52	2.76	31.22	8.88
3	Benzaldehyde	MS, RI, Std	1.0630	0.0355	0.9955	461.73a	9.59	462.78a	5.74	211.13b	6.80	0.86–2.77	320	1.44	1.45	0.66
4	(E, E)-2,4-heptadienal	MS, RI, Std	0.5483	−0.8142	0.9892	25.79	1.59	29.68	2.58	-	-	0.14–0.26	56	0.46	0.53	-
5	Furfural	MS, RI, Std	0.9657	0.0097	0.9934	263.06b	8.69	232.60b	6.85	643.71a	9.62	0.17–0.39	9.56	27.52	24.33	67.33
6	Hexanal	MS, RI, Std	0.2252	0.0112	0.9994	75.49c	6.93	1289.52a	5.14	158.66b	8.10	0.21–1.01	2.4	31.45	537.30	66.11
7	Pentanal	MS, RI, Std	0.0497	0.0036	0.9761	2.12	9.51	22.75	8.72	-	-	0.02–0.17	22	0.10	1.03	-
8	Neral	MS, RI, Std	0.8262	0.0128	0.8953	45.30	8.29	13.76	3.96	-	-	0.15–0.52	-	-	-	-
9	5-Methyl furfural	MS, RI, Std	1.0479	0.0142	0.9545	14.75b	9.46	14.81b	6.87	15.83a	2.64	0.07–0.20	500	0.03	0.03	0.03
10	4-Methoxybenzaldehyde	MS, RI, Std	0.4058	−0.5701	0.9833	-	-	-	-	23.15	0.08	0.05–0.09	-	-	-	-
11	2-Methylbutanal	MS, RI, Std	0.0555	0.0613	0.9919	24.94c	5.11	56.39a	9.61	37.11b	8.67	0.35–0.74	1.5	16.63	37.59	24.74
12	3-Methylbutanal	MS, RI, Std	0.0637	0.0755	0.9931	16.14b	7.41	44.69a	5.20	9.73c	1.11	0.33–0.58	0.5	32.28	89.38	19.46
13	2-Methylpropanal	MS, RI, Std	0.3738	0.0043	0.9946	5.84b	2.31	9.26a	2.33	9.31a	3.65	0.06–0.11	1.9	3.07	4.87	4.90
14	beta-Cyclocitral	MS, RI, Std	0.0861	0.0757	0.9158	2.67	6.68	20.71	8.43	-	-	0.40–0.45	3	0.89	6.90	-
15	(1R)-(-)-Myrtenal	MS, RI, Std	0.0287	0.0025	0.8917	3.07	4.73	-	-	-	-	0.01–0.05	-	-	-	-
16	2-Phenyl-2-butenal	MS, RI, Std	0.1124	−0.0531	0.9790	11.03b	2.54	21.15a	8.37	11.91b	2.67	0.06–0.22	-	-	-	-
Acids																
1	Benzoic acid	MS, RI, Std	0.0571	0.0252	0.9752	29.50b	7.25	96.92a	7.80	27.23b	6.23	0.31–0.51	-	-	-	-
2	Geranic acid	MS, RI, Std	1.0845	0.0032	0.9473	2932.64a	6.37	920.64b	8.00	89.83c	7.29	0.14–1.25	-	-	-	-
3	(E)-2-Hexenoic acid	MS, RI, Std	0.9934	0.0927	0.9072	1479.40b	8.12	2407.72a	6.99	735.78c	2.20	1.46–2.86	1900	0.78	1.27	0.39
4	(E)-3-Hexenoic acid	MS, RI, Std	1.2934	0.0812	0.9137	565.33b	8.65	1770.08a	7.86	281.13c	9.06	0.57–2.68	-	-	-	-
5	Hexanoic acid	MS, RI, Std	0.8850	0.2482	0.9697	740.41b	3.62	4676.66a	6.00	739.90b	8.04	1.47–2.77	1000	0.74	4.68	0.74
6	Butanoic acid	MS, RI, Std	0.3587	0.0226	0.9224	-	-	174.62	4.68	-	-	0.19–0.25	1000	-	0.17	-
Esters																
1	Methyl salicylate	MS, RI, Std	1.4006	0.0029	0.9903	1510.74a	5.93	657.15b	3.00	280.81c	4.36	0.88–3.80	75 *	20.25	8.81	3.76
2	Dihydroactinidiolide	MS, RI, Std	1.0118	0.0028	0.9744	70.60	7.70	387.87	6.78	-	-	0.06–0.38	-	-	-	-
3	Methyl hexanoate	MS, RI, Std	0.1052	−0.2107	0.9092	-	-	68.30	5.58	-	-	0.50–0.60	10	-	6.83	-
4	Benzyl acetate	MS, RI, Std	0.5222	−0.0229	0.9169	1.16	1.41	1.82	5.01	-	-	0.03–0.08	30	0.04	0.06	-
5	γ-Butyrolactone	MS, RI, Std	1.2151	0.0541	0.9505	213.45b	7.08	327.91a	9.82	232.80b	8.92	0.44–0.81	50	4.27	6.56	4.66
Ketones																
1	6-Methyl-5-hepten-2-one	MS, RI, Std	0.2728	−0.0473	0.9973	20.20a	3.58	19.49a	8.70	3.66b	2.85	0.06–0.70	160	0.13	0.12	0.02
2	α-Ionone	MS, RI, Std	0.3657	0.0098	0.9232	-	-	15.43	6.81	-	-	0.80–0.85	58 *	-	0.27	-
3	β-Ionone	MS, RI, Std	0.2648	0.0059	0.9893	2.88b	4.19	29.73a	3.66	3.73b	1.98	0.13–1.14	21 *	0.14	1.42	0.18
4	3-methylnonane-2,4-dione	MS, RI, Std	0.7542	0.0083	0.9063	-	-	0.39	0.19	0.27	0.19	0.27–0.39	0.01	-	39	27
5	β-Damascenone	MS, RI, Std	0.9470	0.0058	0.9896	-	-	0.94	0.11	-	-	0.81–0.98	0.004	-	235	-
6	cis-Jasmone	MS, RI, Std	0.1271	−0.0855	0.9191	16.28	0.56	27.19	6.33	-	-	0.10–0.30	24 *	0.68	1.13	-
7	Coumarin	MS, RI, Std	1.5255	−0.0055	0.8922	86.64	4.89	85.92	2.58	-	-	0.06–0.08	-	-	-	-
8	(E, E)- 3,5-Octadien-2-one	MS, RI, Std	1.0002	0.0043	0.9328	16.42	2.41	10.22	3.85	-	-	0.19–0.31	-	-	-	-
9	2-Heptanone	MS, RI, Std	0.1109	−0.0369	0.9251	-	-	27.64	8.53	-	-	0.04–0.35	0.14	-	197.43	-
10	Isophorone	MS, RI, Std	0.0283	−0.1075	0.8964	-	-	83.05	1.62	-	-	0.09	-	-	-	-
Hydrocarbons																
1	β-Ocimene	MS, RI, Std	0.0621	0.0318	0.9371	403.47a	8.63	223.03b	1.40	15.75c	4.51	0.46–2.67	48 *	8.41	4.65	0.33
2	β-Myrcene	MS, RI, Std	0.3013	−0.0235	0.9127	1.93b	0.49	2.03b	1.44	13.49a	5.99	0.05–0.90	1.2	1.61	1.69	11.24
3	D-Limonene	MS, RI, Std	0.5907	−0.0321	0.9760	31.31a	6.11	19.55b	2.84	6.21c	1.98	1.10–1.73	200	0.16	0.10	0.03
4	Styrene	MS, RI, Std	1.2238	0.0229	0.9075	144.25b	8.97	5.42c	7.63	389.51a	9.65	0.14–0.77	50	2.89	0.11	7.79
5	Longifolene	MS, RI, Std	0.6833	0.0093	0.8994	97.74	6.90	-	-	10.23	4.96	0.12	-	-	-	-
6	Naphthalene	MS, RI, Std	0.8424	0.0047	0.8826	14.50c	5.27	41.43a	8.26	24.33b	6.84	0.10–0.25	-	-	-	-
Sulfide																
1	Dimethyl sulfide	FPD, RI, Std	0.5279	0.0034	0.9957	25.95b	2.98	20.64b	1.74	42.68a	4.87	0.23–0.51	0.84	30.89	24.57	50.81
2	Dimethyl disulfide	FPD, RI, Std	0.0685	−0.0051	0.9781	-	-	-	-	2.76	0.40	0.03	1.1	-	-	2.51
Pyrazines																
1	2-Methylpyrazine	MS, RI, Std	1.2058	0.0062	0.9606	202.00a	8.50	33.93c	5.50	129.79b	4.63	0.08–0.24	60	3.37	0.57	2.16
2	2,6-Dimethylpyrazine	MS, RI, Std	0.9318	0.0003	0.9502	64.04	4.88	-	-	-	-	0.04	6	10.67	-	-
3	2-Ethyl-pyrazine	MS, RI, Std	1.3661	0.0048	0.9125	9.89	2.94	-	-	8.92	9.08	0.03–0.08	4	2.47	-	2.23
Others																
1	1-Ethyl-1H-pyrrole-2-carbaldehyde	MS, RI, Std	1.0876	0.0128	0.9878	761.04b	9.17	371.11c	8.01	2012.45a	3.88	0.35–0.61	-	-	-	-
2	2-Formyl-1H-pyrrole	MS, RI, Std	1.0270	0.0168	0.9002	950.35a	8.62	564.26b	5.10	977.42a	6.61	0.65–0.75	-	-	-	-
3	2-Acetyl pyrrole	MS, RI, Std	1.0799	0.0515	0.9831	1123.74a	4.19	181.36c	8.94	927.73b	8.72	0.53–1.08	-	-	-	-
4	2-Ethylfuran	MS, RI, Std	0.0662	0.0107	0.9464	4.98b	2.24	16.09a	4.82	1.99c	0.35	0.07–0.18	100	0.05	0.16	0.02
5	2-Pentylfuran	MS, RI, Std	0.9381	−0.6939	0.9097	12.82b	0.46	15.64a	2.69	12.40b	0.25	0.07–0.26	4.8	2.67	3.26	2.58

^a^ OTs, odor threshold in water; the data with * are obtained in this study; other data comes from the Leffingwell & Associates and the literature [18,19,20]; ^b^ values with different superscript roman letters (a–c) in the same row are significantly different according to the Duncan test (*p* < 0.05); ^c^ -, not detected.

**Table 3:** The n value of Steven’s law and the n difference.

Compounds	Concentration (μg/kg)	Intensity ^a^	Iteration Number ^b^	K ^c^	N ^d^	RSS ^e^	Steven’s Value	n_MeSA_−n	(n_MeSA_ + n)/2	N
MeSA	800	1.85	12.1	0.007	0.800	2.153	1.89	-	-	-
	…^f^	…		0.031	0.615	0.562	…			
	6400	6.50					6.79			
Linalool	1000	4.17	4.1	0.904	0.236	0.861	4.78	0.402	0.414	0.287
	…	…		1.098	0.213	0.757	…			
	16,000	8.40					8.63			
Phenylacetaldehyde	1000	4.90	6.1	0.462	0.335	1.285	4.90	0.320	0.455	0.226
	…	…		0.638	0.295	1.003	…			
	8000	8.67					9.04			
Geraniol	50	4.63	4.1	1.300	0.400	0.732	4.47	0.308	0.461	0.269
	…	…		1.345	0.307	0.57	…			
	400	8.30					8.46			
Phenethyl alcohol	750	1.50	4.1	0.193	0.328	0.245	1.76	0.312	0.459	0.445
	…	…		0.237	0.303	0.222	…			
	12,000	4.13					4.08			
β-Ionone	20	3.10	4.1	1.762	0.206	0.159	3.33	0.421	0.405	0.358
	…	…		1.861	0.194	0.144	…			
	320	5.60					5.70			
cis-Jasmone	50	0.87	6.1	0.151	0.467	0.256	1.05	0.212	0.509	0.693
	…	…		0.218	0.403	0.182	…			
	800	3.03					3.22			
Nerol	800	1.48	11.1	0.010	0.744	1.241	1.80	0.012	0.609	0.815
	…	…		0.032	0.603	0.595	…			
	6400	6.00					6.31			
Benzyl alcohol	20,000	1.13	9.1	0.004	0.597	2.169	1.54	0.096	0.567	0.684
	…	…		0.009	0.519	0.241	…			
	32,0000	6.40					6.48			
α-terpineol	8000	1.63	19.1	0.000	0.400	75.945	1.78	0.039	0.635	0.700
	…	…		0.005	0.654	0.716	…			
	64,000	6.55					6.95			
β-ocimene	100	3.00	9.1	0.112	0.650	4.685	2.81	0.154	0.538	0.785
	…	…		0.336	0.461	1.798	…			
	800	6.93					7.32			

^a^ Average intensity value obtained by the sensory panel member; ^b^ iteration number means major iteration and minor iteration; ^c^ initial k value and optimal k value; ^d^ initial n value and optimal n value; ^e^ RSS, residual sum of squares; ^f^ …, represented the corresponding relationship for concentration, aroma intensity and Steven’s value of each compound (10 groups).

## Data Availability

The data that support the findings of this study are available from the corresponding author, W.X., upon reasonable request.

## Supplementary Materials

Figure S1: TICs for HS-SPME and SAFE for the JJM sample; Table S1: Supplementary information for HS-SPME/SAFE quantification.

## Sample Availability

Samples of the compounds are available from the authors.
