# Supplementary material for: Characterization of the Key Aroma Compounds of Three Kinds of Chinese Representative Black Tea and Elucidation of the Perceptual Interactions of Methyl Salicylate and Floral Odorants
Source: Molecules. 2022 Mar 1;27(5):1631. doi: 10.3390/molecules27051631 (PMC8911931; doi:10.3390/molecules27051631)
Supplement: Supplementary file 1 [file molecules-27-01631-s001.zip › molecules-1607696-supplementary.pdf]

Supplementary materials:

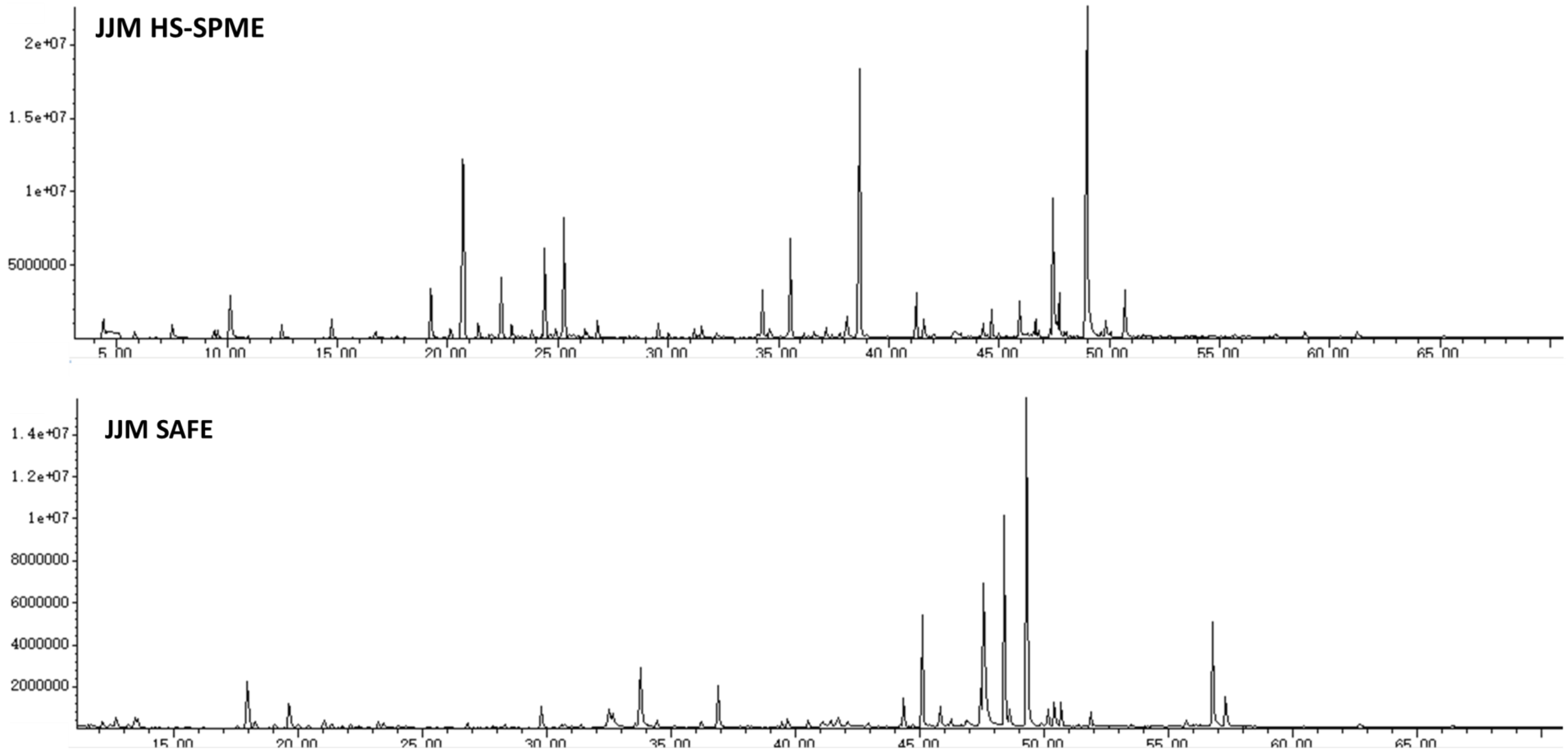

Figure S1. TICs for HS-SPME and SAFE for the JJM sample

Table S1. Supplementary information for HS-SPME/SAFE quantification.

No.	Odorant	HS-SPME		Validation Range (μg/kg)	SAFE		Validation Range (μg/kg)
		LOD[a](μg/kg)	LOQ[b](μg/kg)		LOD(μg/kg)	LOQ(μg/kg)	
Alcohols							
1	Linalool	0.01	0.03	450-7000	0.01	0.03	200-6000
2	Geraniol	0.01	0.03	200-8000	0.02	0.06	600-6000
3	Phenylethyl alcohol	0.10	0.30	100-10000	0.20	0.60	1000-25000
4	(Z)-3-Hexenol	0.46	1.38	50-1000	0.01	0.03	200-2500
5	Benzyl alcohol	0.10	0.30	500-10000	0.10	0.30	200-15000
6	*cis* -Linaloloxide	0.02	0.06	100-1000	0.15	0.45	50-500
7	Nerol	0.02	0.06	100-1000	0.03	0.10	50-500
8	α-Terpineol	0.03	0.09	10-100	0.10	0.30	1-50
9	1-Hexanol	0.05	0.15	1-50	0.60	1.80	0.5-20
10	*trans*-Linaloloxide	0.01	0.03	50-500	0.20	0.60	5-200
11	α-Methylbenzyl alcohol	0.03	0.09	1-100	0.25	0.75	5-100
12	2-Pentenol	0.20	0.60	50-200	0.10	0.30	100-200
13	1-Penten-3-ol	0.10	0.30	50-500	0.25	0.75	50-500
14	4-Terpinenol	0.05	0.15	10-50	0.20	0.60	2-20
15	2-Ethyl-1-hexanol	0.20	0.60	100-500	0.10	0.30	50-500
16	Furfuryl alcohol	1.20	3.60	50-500	1.15	3.45	150-1500
17	1-Octen-3-ol	0.22	0.66	5-200	1.06	3.18	20-200
18	Hotrienol	0.50	1.50	20-500	1.85	5.55	50-200
Aldehydes							
1	(E)-2-Hexenal	0.15	0.45	100-500	0.65	1.95	200-500
2	Benzeneacetaldehyde	0.10	0.30	500-2500	0.11	0.33	100-2000
3	Benzaldehyde	0.17	0.51	50-200	0.20	0.60	200-500
4	(E, E)-2,4-Heptadienal	0.08	0.24	2-50	0.78	2.34	1-50
5	2-Methyl-Butanal	0.30	0.90	20-100	-[c]	-[c]	-
6	3-Methyl-Butanal	0.50	1.50	5-50	-[c]	-[c]	-
7	2-Methylpropanal	0.10	0.30	1-10	-[c]	-[c]	-
8	Furfural	0.03	0.09	100-1000	1.40	4.21	200-1000
9	Hexanal	0.01	0.03	50-1500	0.06	0.18	100-1000
10	Pentanal	0.20	0.60	2-50	0.40	1.20	5-50
11	Neral	0.10	0.30	2-50	0.50	1.50	5-50
12	5-Methyl furfural	0.20	0.60	5-50	0.65	1.95	5-20
13	4-Methoxybenzaldehyde	0.50	1.50	5-50	2.15	3.45	10-50

14	beta-Cyclocitral	0.20	0.60	5-50	3.76	11.28	ND[d]
15	(1R)-(-)-Myrtenal	0.02	0.06	1-5	4.96	14.88	ND
16	2-Phenyl-2-Butenal	0.40	1.20	10-50	3.05	9.15	ND
Acids							
1	Benzoic acid	1.15	3.45	20-100	1.09	3.27	50-150
2	Geranic acid	0.65	1.95	100-2000	0.88	2.64	50-3000
3	(E)-2-Hexenoic acid	0.46	1.38	500-2000	0.55	1.65	500-2500
4	(E)-3-Hexenoic acid	0.12	0.36	500-2000	0.39	1.47	200-2000
5	Hexanoic acid	0.06	0.18	200-5000	0.46	1.38	500-5000
6	Butanoic acid	5.62	16.86	50-200	2.16	6.48	20-200
Esters							
1	Methyl salicylate	0.14	0.42	150-1500	0.11	0.33	200-2000
2	Dihydroactinidiolide	7.76	23.28	ND	1.07	3.21	10-500
3	Methyl hexanoate	0.04	0.12	20-100	0.10	0.30	10-100
4	Benzyl acetate	0.03	0.09	0.5-2	0.12	0.36	1-5
5	γ-Butyrolactone	3.25	9.75	ND	0.53	1.59	10-500
Ketones							
1	6-Methyl-5-hepten-2-one	0.06	0.18	2-50	0.22	0.66	5-50
2	α-Ionone	0.20	0.60	2-20	1.49	4.47	ND
3	β-Ionone	0.10	0.30	1-50	1.56	4.68	ND
4	3-methylnonane-2,4-dione	0.03	0.09	0.2-10	0.05	0.15	1-20
5	β-Damascenone	0.04	0.12	0.5-10	0.13	0.39	1-20
6	cis-Jasmone	0.25	0.75	2-50	2.88	8.64	ND
7	Coumarin	6.76	20.28	ND	0.70	2.10	10-100
8	(E, E)-3,5-Octadien-2-one	0.32	0.96	2-20	0.51	1.53	5-50
9	2-Heptanone	0.16	0.48	5-50	0.54	1.62	5-50
10	Isophorone	0.47	1.41	5-100	3.45	10.35	ND
Hydrocarbons							
1	β-Ocimene	0.05	0.15	10-500	0.03	0.09	50-500
2	β-Myrcene	0.04	0.12	1-20	0.02	0.06	5-20
3	D-Limonene	0.04	0.12	5-50	0.04	0.13	10-50
4	Styrene	0.04	0.12	2-500	0.10	0.30	5-200
5	Longifolene	0.24	0.72	10-100	0.46	1.38	50-100
6	Naphthalene	0.31	0.93	10-50	1.15	3.45	20-100
Sulfide							

1	Dimethyl sulfide	0.10	0.30	10-50	-[c]	-[c]	-
2	Dimethyl disulfide	0.10	0.30	1-20	-[c]	-[c]	-
Pyrazines							
1	2-Methylpyrazine	5.71	17.13	ND	0.10	0.30	10-200
2	2,6-Dimethyl-pyrazine	7.60	22.80	ND	0.50	1.50	10-100
3	2-Ethyl-pyrazine	1.92	5.76	10-20	0.35	1.05	5-20
Others							
1	1-Ethyl-1H-pyrrole-2-carbaldehyde	0.72	2.16	200-2500	2.02	6.06	500-2000
2	2-Formyl-1H-pyrrole	0.66	1.98	500-1000	1.94	5.82	500-1000
3	2-Acetyl pyrrole	0.79	2.37	100-1500	2.49	7.47	200-1000
4	2-Ethyl-furan	0.50	1.50	5-20	0.10	0.30	1-20
5	2-Pentyl-furan	0.61	1.83	10-20	0.40	1.20	5-20

[a] LOD: Limits of detection (LOD). [b] LOQ: Limits of quantitation (LOQ). [c] -: The compound was not detected due to the solvent delay of SAFE. [d] ND: Not detected.
